# Corticospinal Tract Injury Precedes Thalamic Volume Reduction in Preterm Infants with Cystic Periventricular Leukomalacia

**DOI:** 10.1016/j.jpeds.2015.05.013

**Published:** 2015-08

**Authors:** Karina J. Kersbergen, Linda S. de Vries, Floris Groenendaal, Ingrid C. van Haastert, Andrew T.M. Chew, Antonios Makropoulos, Sarah L. Dawson, Frances M. Cowan, Manon J.N.L. Benders, Serena J. Counsell

**Affiliations:** 1 Department of Perinatology, Wilhelmina Children's Hospital and Brain Center Rudolf Magnus, University Medical Center Utrecht, Utrecht, The Netherlands; 2 Center for the Developing Brain, Division of Imaging Sciences and Biomedical Engineering, King's College London, London, United Kingdom; 3 Biomedical Image Analysis Group, Department of Computing, Imperial College London, London, United Kingdom; 4 Department of Pediatrics, Hammersmith Campus, Imperial College London, London, United Kingdom

**Keywords:** AD, Axial diffusivity, AUC, Area under the curve, CP, Cerebral palsy, c-PVL, Cystic periventricular leukomalacia, CST, Corticospinal tract, DTI, Diffusion tensor imaging, FA, Fractional anisotropy, GA, Gestational age, GMFCS, Gross motor function classification system, MD, Mean diffusivity, MRI, Magnetic resonance imaging, NPV, Negative predictive value, PLIC, Posterior limb of the internal capsule, PMA, Postmenstrual age, PPV, Positive predictive value, PVL, Periventricular leukomalacia, RD, Radial diffusivity, TBV, Total brain volume, TE, Echo time, TEA, Term equivalent age, TR, Repetition time

## Abstract

**Objectives:**

To measure both fractional anisotropy (FA) values in the corticospinal tracts (CSTs) and volume of the thalami in preterm infants with cystic periventricular leukomalacia (c-PVL) and to compare these measurements with control infants.

**Study design:**

Preterm infants with c-PVL and controls with magnetic resonance imaging data acquired between birth and term equivalent age (TEA) were retrospectively identified in 2 centers. Tractography of the CST and segmentation of the thalamus were performed, and values from infants with c-PVL and controls were compared.

**Results:**

Thirty-three subjects with c-PVL and 31 preterm controls were identified. All had at least 1 scan up to TEA, and multiple scans were performed in 31 infants. A significant difference in FA values of the CST was found between cases and controls on the scans both before and at TEA. Absolute thalamic volumes were significantly reduced at TEA but not on the earlier scans. Data acquired in infancy showed lower FA values in infants with c-PVL.

**Conclusions:**

Damage to the CST can be identified on the early scan and persists, whereas the changes in thalamic volume develop in the weeks between the early and term equivalent magnetic resonance imaging. This may reflect the difference between acute and remote effects of the extensive injury to the white matter caused by c-PVL.

Despite the decreasing incidence, cystic periventricular leukomalacia (c-PVL) remains one of the leading causes of cerebral palsy (CP) after preterm birth.^1,2^ Around 1%-3% of preterm infants will have c-PVL,^1-3^ and in over 60% of them, this will develop CP.^2,4^ Early recognition of infants at risk of CP is important, both for accurate counseling of parents as well as for a possible selection of infants that may benefit from early behavioral interventions or rehabilitation services.^5^ Assessment of the myelination of the posterior limb of the internal capsule (PLIC) on conventional magnetic resonance imaging (MRI) at term equivalent age (TEA) can reliably predict the development of CP in most infants.^6^ However, the severity of CP as well as cognitive and behavioral outcomes and the development of epilepsy, remain difficult to predict.

Over the last decade, the use of diffusion tensor imaging (DTI) has made the in vivo assessment of early human brain development possible on a microstructural level, providing a better understanding of both the direct and remote effects of injury to white or gray matter.^7^

Several studies have shown a correlation between the presence and severity of CP after periventricular leukomalacia (PVL) and fractional anisotropy (FA) in the corticospinal tract (CST) as a measure of white matter injury in childhood. FA measurements were lower in children with CP compared with healthy controls, and also lower in children who were more severely impaired compared with those with mild, ambulatory CP.^8-10^ It is, however, unclear when these differences develop because these studies were performed in older children. Studies in the neonatal period are few, and often include infants with non-c-PVL, which may influence the results.^11^ This is also the case for neonatal studies assessing thalamic involvement in children with CP after PVL. Thalamic involvement is thought to occur as a secondary process to white matter injury, and thalamic volumes were reduced in children with CP compared with healthy controls.^12,13^ Again, this appears to be related to the severity of CP in childhood,^14,15^ but it is unclear when these differences develop.

The aim of our study was to test the hypothesis that FA values in the CST and thalamic volumes in preterm infants with c-PVL, scanned during the early neonatal period and again around TEA, are lower compared with these measurements in control infants. In addition, we aimed to assess the association between these measures and gross motor outcome in infancy.

## Methods

For this study, all infants born below a gestational age (GA) of 36 weeks with a clinical diagnosis of c-PVL on cranial ultrasound and at least 1 MRI during the neonatal period were retrospectively identified. Infants born between March 2005 and September 2013 from both the Wilhelmina Children's Hospital in Utrecht, The Netherlands and Queen Charlotte's and Chelsea Hospital in London, United Kingdom, were eligible for inclusion. Permission for MRI was granted by the Research Ethics Committees at both institutions. PVL grading was determined on sequential cranial ultrasound, according to de Vries et al.^16^ Scans were obtained within the first few weeks after the insult occurred as assessed from cranial ultrasound scans in most patients and were repeated at TEA. Preterm control infants without significant brain injury on their MRI and normal motor development at follow-up around 15 months corrected age were randomly selected from our preterm population and were matched for sex. Scans in the controls were done around 30 weeks postmenstrual age (PMA) for infants with a GA <28 weeks and soon after birth on clinical indication in those with a GA >28 weeks, and were repeated at TEA. For a subgroup of patients with c-PVL, scans in infancy were also available (n = 10, 5 from Utrecht, 4 from London, with 1 of the infants from London scanned twice). Three control infants from London were scanned in infancy, and no control scans during infancy from Utrecht were available. Outcome data were collected from the patient charts. Neurodevelopmental outcome was formally assessed in the out-patient follow-up clinic, and all children had a standard clinical neurologic examination. Severity of CP was graded according to the gross motor function classification system (GMFCS).^17^

### MRI

For the infants from Utrecht, MRI was performed on either a 1.5 Tesla ACS-NT system or a 3 Tesla whole-body Achieva system (Philips Medical Systems, Best, The Netherlands) with the phased array head coil. On the 1.5T magnet, the routine protocol included conventional inversion recovery-weighted imaging and T2-weighted imaging (30 and 40 weeks: inversion recovery-weighted repetition time [TR] 4147 ms; inversion time 600 ms; echo time [TE] 30 ms; slice thickness 2 mm and T2-weighted TR 7656 ms; TE 150 ms; slice thickness 2 mm) and on the 3T magnet, the routine protocol included conventional 3-dimensional T1-weighted and T2-weighted imaging (30 weeks: 3-dimensional T1-weighted TR 9.4 ms; TE 4.6 ms; slice thickness 2 mm and T2-weighted TR 10 085 ms; TE 120 ms; slice thickness 2 mm; 40 weeks: 3D T1-weighted TR 9.5 ms; TE 4.6 ms; slice thickness 1.2-2 mm and T2-weighted TR 4847-6293 ms; TE 120-150 ms; slice thickness 1.2-2 mm). The DTI protocol consisted of a single-shot spin-echo echo-planar imaging sequence (echo-planar imaging factor 55, TR 5685 ms, TE 70 ms, field of view 180 × 146 mm, acquisition matrix 128 × 102 mm (voxel size 1.41 × 1.43 × 2.0 mm), 2 mm slice thickness without gap, SENSE factor 2). Images were acquired in the axial plane with diffusion gradients applied in 32 noncollinear directions with a b-value of 800 s/mm^2^ and 1 nondiffusion-weighted image. In infancy, all children were scanned on a 1.5T magnet. A similar single-shot echo-planar imaging sequence was used but with an echo-planar imaging factor of 51, TR of 8382 ms, and field of view of 192 × 192 mm. Infants from London were scanned on a Philips 3 Tesla system (Philips Medical Systems, Best, The Netherlands) using an 8-channel phased array head coil. T2-weighted fast-spin echo MRI was acquired using TR 8670 ms; TE 160 ms; slice thickness 2 mm with 1 mm overlapping slices and T1-weighted imaging using TR 17 ms, TE 4.6 ms, slice thickness 0.8 mm. Single shot echo-planar imaging DTI was acquired in the transverse plane in 32 noncollinear directions using the following variables: echo planar imaging factor 59, TR 8000 ms, TE 49 ms, field of view 224 mm, acquisition matrix 128 × 128 (voxel size 1.75 × 1.75 × 2.0 mm), 2 mm slice thickness, b-value 750 s/mm^2^, SENSE factor of 2.

Infants in Utrecht were sedated for the first 2 scans, using oral chloral hydrate 50-60 mg/kg, and in London only for the TEA scan (30-55 mg/kg), according to the local clinical protocol. A neonatologist was present throughout all examinations. In infancy, children in Utrecht received general anesthesia and were monitored by an anesthetist, and those in London were sedated with oral chloral hydrate (50-80 mg/kg) and monitored by an experienced pediatrician.

For all MRIs obtained around TEA, the presence and quality of myelination in the PLIC were scored retrospectively (F.C., L.d.V) as either normal or abnormal/absent, using both T1- and T2-weighted sequences. Scoring was performed blinded to outcome data.

### Postprocessing of Volumetric Data

**Figure 1:**
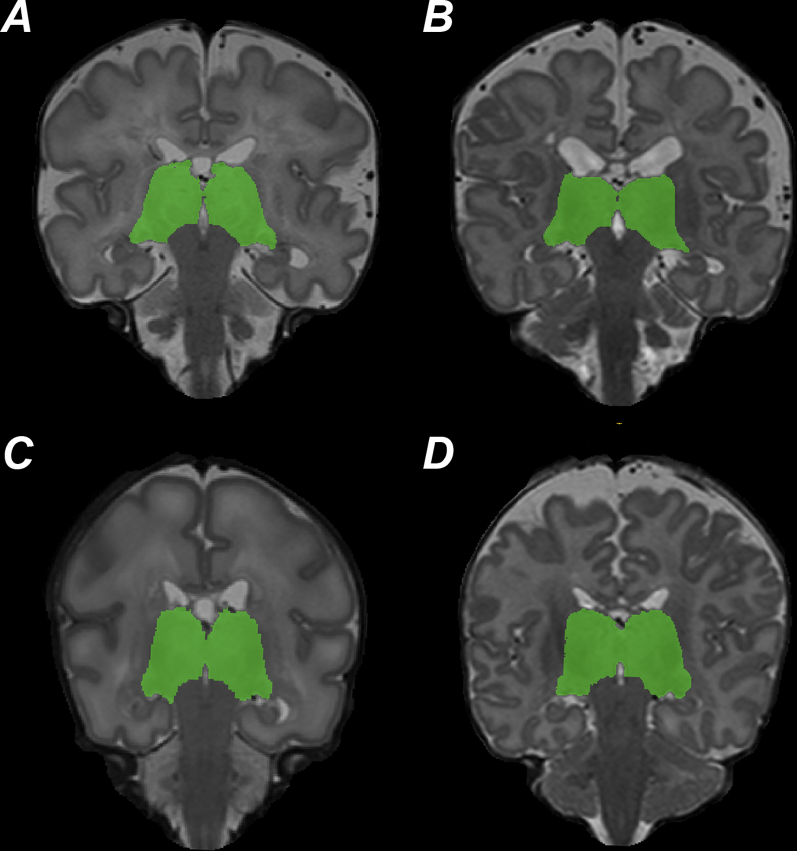
Thalamic segmentations. The upper row represents a male infant, born at 32 weeks GA and imaged at **A,** 33 weeks and **B,** 40+3 weeks. Note the low signal intensity lesions in the periventricular white matter on the early T2-weighted image and the periventricular cysts with enlarged ventricles at TEA. The lower row represents a female infant, born at 27+6 weeks and imaged **C,** at 30+3 and **D,** 41+3 weeks. No evidence of brain injury was observed on her scan.

T2-weighted scans during the neonatal period were segmented using the neonatal specific segmentation method of Makropoulos et al.^18^ This method utilizes an expectation-maximization scheme that combines manually labeled atlases^19^ with intensity information from the image to be segmented and has shown reliable results among infants scanned at PMA ranging between 28 and 44 weeks.^18,20^ All segmentations were manually checked, and small corrections were performed if necessary. An example of segmentation results in cases and controls is presented in Figure 1 (available at www.jpeds.com).

For the infants scanned again in infancy, not all data were acquired at high resolution and, therefore, not all T2-data were suitable for accurate segmentation. The data of the infants with high resolution imaging (n = 6) were segmented using an atlas and segmentation approach specifically designed for infants and thalamic volumes in infancy were calculated for these 6 infants.^21^ Bilateral thalamic volumes and total brain volume (TBV) were extracted for all scans.

### Postprocessing of DTI Data

**Figure 2:**
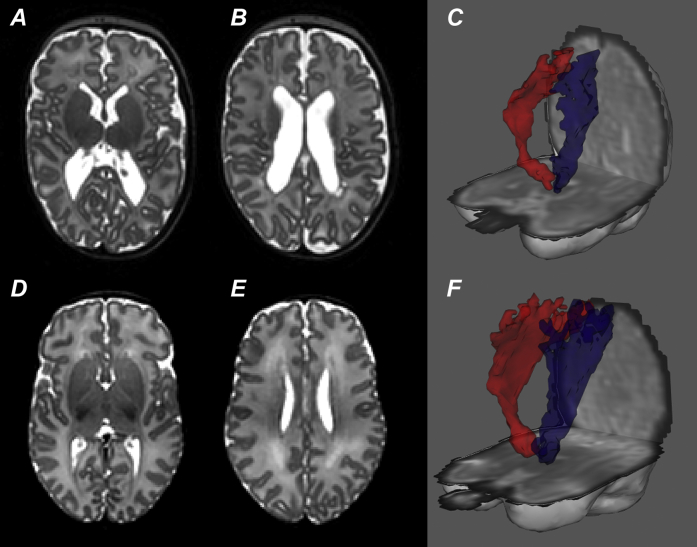
Axial T2 weighted images at the level of the **A,** basal ganglia and **B,** midventricular level in a male infant, born at 30 weeks GA and imaged at 40 weeks, with c-PVL. The **A,** basal ganglia are small, the ventricles are dilated, and **B,** cystic lesions can be seen posterior to the posterior horns of the lateral ventricles. **C,** shows 3D representations of the CSTs. Axial T2 weighted images at the level of the **D,** basal ganglia and **E,** midventricular level in a female infant, born at 30+5 weeks GA and imaged at 39+6 weeks with no evidence of lesions on MRI. **F,** shows 3D representations of the CSTs. *3D*, 3-dimensional.

DTI data were analyzed using the Oxford Centre for Functional Magnetic Resonance Imaging of the Brain Software Library.^22^ Probabilistic tractography of the bilateral CST was performed as described previously.^23^ A seed mask (early scan 17 voxels, TEA scan 20 voxels, late scan 24 voxels) was drawn in the cerebral peduncle, with waypoint masks in the PLIC (early scan 17 voxels, TEA 21 voxels, late scan 24 voxels) and around the central sulcus. Connectivity distributions were generated from every voxel in the seed masks, and only those paths that went through the waypoint masks were retained. The tracts were normalized by the number of samples going from the seed mask through the waypoints.^24^ These connectivity distributions were then thresholded at probability >1%. An example of the resulting tracts for both cases and controls can be found in Figure 2 (available at www.jpeds.com). Corresponding FA, and mean diffusivity (MD), axial diffusivity (AD), and radial diffusivity (RD) values were subsequently calculated for both the entire CST and the PLIC mask itself.

### Statistical Analyses

Statistical procedures were performed using R v 2.15.3 (R Foundation for Statistical Computing, Vienna, Austria).^25^ FA values and thalamic volumes from infants with c-PVL and controls, as well as the relation between FA and volumetric measurements and both PLIC scoring (defined as normal or abnormal) and outcome for the infants with c-PVL, were compared using multiple linear regression modeling. PMA at time of scan and the interaction between the FA value or volume and PMA were included in the model. Because FA values can differ per DTI-protocol, only infants and controls scanned with the same protocol were included in the statistical analysis, and data from Utrecht and London were analyzed separately. Subsequent analysis of the combined data was performed with center included as a covariate in the model; 95% CIs for the figures were calculated using GraphPad Prism v 6.05 software (GraphPad Software Inc, La Jolla, California). Left-right differences were tested with paired-samples *t* tests. The relation between the PLIC scoring and outcome was calculated with a Fisher exact test. Sensitivity, specificity, positive predictive value (PPV), and negative predictive value (NPV) of the different measurements with regards to outcome were assessed by receiver operating characteristic curves. The area under the curve (AUC) was used to determine the optimal cut-off point. As no significant left-right differences were found, data were grouped for this analysis.

## Results

Thirty-three infants, 26 from Utrecht and 7 from London, with c-PVL were eligible for inclusion. The majority of patients had grade III c-PVL (23 from Utrecht, 5 from London).^16^ Two patients from Utrecht and 2 from London had grade II c-PVL and 1 patient from Utrecht had widespread precystic lesions but died before these could evolve into cysts. Lesion appearance was largely symmetrical. Clinical data on the included infants are presented in Table I. An overview of the number of available scans and the extent of serial imaging data are shown in Table II (available at www.jpeds.com).

### Survival and Motor Outcome

Three of the 33 infants with c-PVL died during the neonatal period. Four of the infants did not develop CP, and 3 have an evolving motor disorder but are too young to be reliably classified with the GMFCS. The remaining 23 infants all developed CP, which was classified as level I in 4 infants, level II in 2 infants, level III in 10 infants, level IV in 5 infants, and level V in 2 infants. Seven infants with c-PVL developed epilepsy: 5 with CP level IV-V, 1 without CP but with severe visual impairment, and 1 with an evolving motor disorder. None of the control infants developed CP or epilepsy.

### Visual Analysis of the Myelination of the PLIC on Scans at TEA

Of the 33 infants with c-PVL, T1- and T2-weighted imaging at TEA was available in 29. The other 4 infants were either deceased (n = 3), or did not have imaging around TEA (n = 1). Of those 29, 8 had myelination of the PLIC that appeared normal and in 21 infants, the PLIC myelin appeared abnormal (abnormal signal intensity, n = 16; absent signal intensity in the region of the PLIC, n = 5). All control infants had myelin in the PLICs that appeared normal.

GMFCS grading was available for 26 of these 29 case infants and 3, all with abnormally appearing PLICs, were too young for accurate CP classification using the GMFCS. When correlated to outcome at 15 months corrected age and beyond, 7 out of 8 infants with normally appearing PLICs and 3 out of 18 with abnormally appearing PLICs had a favorable outcome (no CP or GMFCS I-II). In contrast, 1 out of 8 infants with normally appearing PLICs and 15 out of 18 infants with abnormal PLICs had an unfavorable outcome (GMFCS III-V), leading to an OR of 35 (95% CI 3.1-399).

No clear correlation between the appearance of the PLIC and diffusivity measurements of the CST or PLIC mask at TEA could be found. A significant correlation between thalamic volumes at TEA and the appearance of the PLICs was found for both the absolute volumes (left *P* = .03, right *P* = .01) and the thalamic volume/TBV ratio (left *P* = .046, right *P* = .02).

When comparing the different measurements at TEA in terms of sensitivity, specificity, PPV, and NPV with regard to outcome, the appearance of the PLIC on conventional MRI was highly predictive (0.94, 0.70, 0.83, and 0.88, respectively). Thalamic volumes were predictive for both absolute (AUC 0.78, optimal cut-off 3450.81, sensitivity 0.64, specificity 0.93, PPV 0.88, and NPV 0.78) and relative volumes (AUC 0.75, optimal cut-off 0.01, sensitivity 0.64, specificity 0.90, PPV 0.82, and NPV 0.77). These values were lower for FA of the CST (AUC 0.58, optimal cut-off 0.23, sensitivity 0.75, specificity 0.50, PPV 0.50, and NPV 0.75 for the Utrecht data, not enough data points to reliably calculate for the London data).

### Corticospinal Tractography

**Figure 3:**
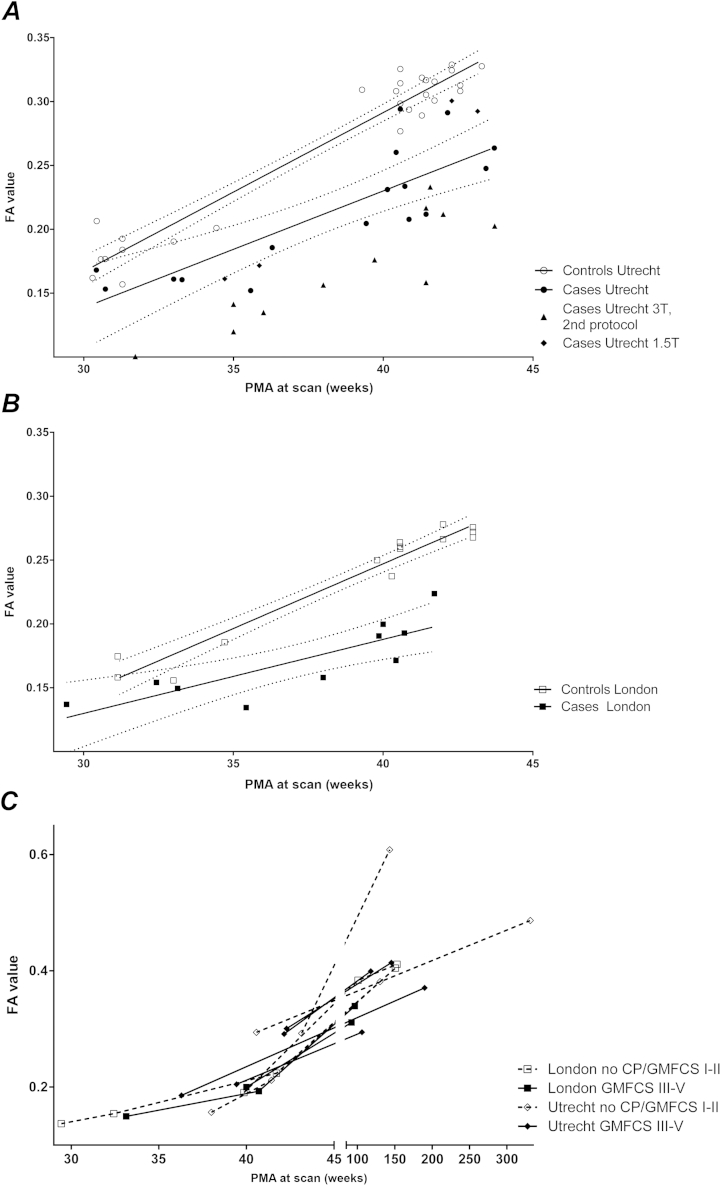
Mean FA values of the left CST for infants with c-PVL and control infants, with dotted lines representing the 95% CIs. Data are shown separately for the infants from **A,** Utrecht and **B,** London. **C,** FA values of the infants with c-PVL scanned again during infancy. Here, the open figures and dotted lines represent the infants with no or ambulatory CP and the solid figures and lines represent the infants with severe CP.

Mean FA values at the different PMAs from the left CST are depicted in Figure 3. No significant left-right differences were found. Because different DTI protocols were used, analyses were performed separately on the London and Utrecht cohorts, and also only between patients and controls tested with the same DTI protocol. However, a similar pattern could be seen for the infants scanned with the different protocols (Figure 3, A). A significant difference was found between patients and controls for both left and right CST when early and TEA scans were combined (London: left *P* = 7.39*10^−4^, right *P* = .011, Utrecht: left *P* = 6.84*10^−10^, right *P* = 8.02*10^−7^). For the left CST of the Utrecht data, the model including the interaction between PMA and the factor case/control was also significant (case/control *P* = .2, interaction *P* = .03). When separated according to time of scanning, these differences persisted except for the right CST of the Utrecht data at the early scan (early scan London: left *P* = .03, right *P* = .03, Utrecht: left *P* = .01, right *P* = .16 and TEA scan London: left *P* = 6.8*10^−7^, right *P* = 9.19*10^−4^, Utrecht: left *P* = 4.3*10^−8^, right *P* = 8.6*10^−6^). When data were pooled and imaging center was added as a covariate, similar results were found, with the differences between cases and controls being significant across all measurements (early scan: left case/control *P* = 5.2*10^−4^, center 6.4*10^−3^, right case/control 6.8*10^−3^, center not significant; TEA scan: left case/control 1.13*10^−13^, center 4.12*10^−10^, right case/control 4.9*10^−9^, center 7.7*10^−8^). Repeating the analysis with AD, MD, and RD values, again with center added as covariate, significant differences between cases and controls were observed for MD measurements at TEA only (left *P* = 5.5*10^−4^, right *P* = .01). This was due primarily to a difference in RD, which was significant at TEA (left *P* = 8.58*10^−6^, right *P* = 7.6*10^−4^). No significant differences in AD values between cases and controls were observed at any time point.

**Figure 4:**
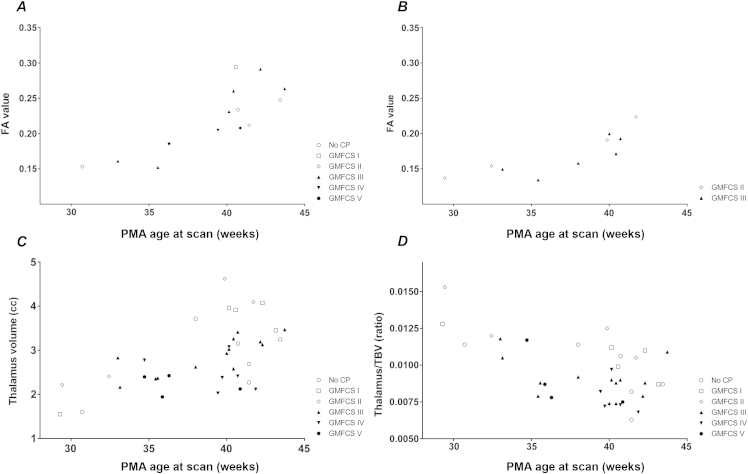
FA values (**A,** Utrecht; **B,** London), **C,** thalamic volumes, and **D,** thalamic/TBV ratios of the infants with c-PVL. Open symbols are used for those infants without CP or with ambulatory CP, and closed symbols for those with nonambulatory CP. For the DTI data, only infants with the 32 directions DTI protocols are shown.

In the infants with serial data, there was an increase in FA for both cases and controls with increasing PMA, similar to the cross-sectional data in Figure 3, A and B. FA increased further after TEA, as shown in the infants with a repeat scan in infancy (Figure 3, C). Figure 4 (available at www.jpeds.com) shows the correlation between FA values at TEA and the severity of CP for each center. No significant differences between FA values at TEA and CP severity were observed, possibly because of the relatively small sample size.

### Diffusivity Measures of the PLIC Mask

In order to assess quantitatively injury to the PLIC, diffusivity measures in the PLIC mask were also measured. Similar to the CST analyses, center was added as covariate. For the Utrecht data, only infants with the 3T 32 directions DTI imaging protocol were included. For the FA values, significant differences between cases and controls were found both at 30 weeks (left *P* = 4.0*10^−4^, right *P* = 5.1*10^−4^) and at TEA (left *P* = 3.34*10^−8^, right *P* = 5.93*10^−6^). MD values did not show any significant differences. AD values differed significantly at 30 weeks for the right side (*P* = .02) and at TEA bilateral (left *P* = 3.03*10^−4^, right *P* = 6.03*10^−4^). RD values were significantly different only at TEA (left *P* = 7.24*10^−5^, right *P* = 7.02*10^−3^).

### Thalamic Volumes

**Figure 5:**
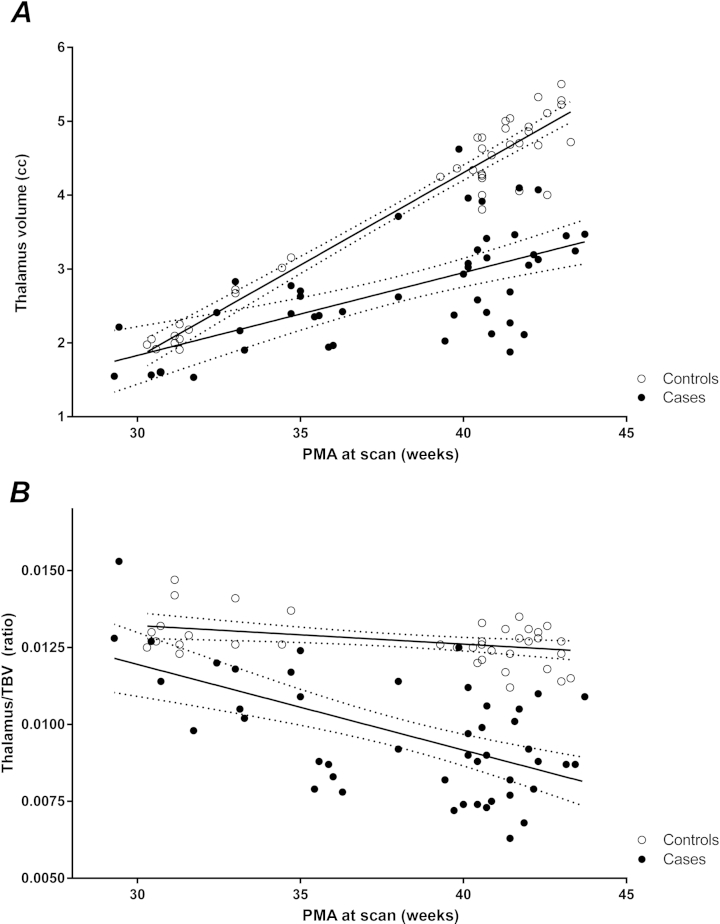
Difference in **A,** absolute and **B,** relative thalamic volumes in infants with c-PVL and control infants. Both centers are shown together. The dotted lines represent the 95% CIs.

Figure 5 shows the thalamic volumes for the left hemisphere, as well as the thalamic volumes corrected for TBV. No significant left-right differences or differences between infants from Utrecht and London were found. Therefore, data from Utrecht and London were combined for the analysis. When volumes for both early and TEA scans were combined, a significant difference was found between cases and controls for both the left and right thalamus, with the interaction between PMA and the factor case/control also being significant (left case/control *P* = 1.3*10^−5^ and interaction *P* = 7.6*10^−8^, right *P* = 3.7*10^−5^ and *P* = 2.8*10^−7^). When separated according to time of scanning, the early data (PMA <34.8 weeks) no longer showed a significant difference. At TEA, the difference between infants with c-PVL and controls was highly significant, and no interaction with PMA was found (left *P* = 1.08*10^−13^ and right *P* = 2.7*10^−13^). After correction for TBV, the combined data showed a correlation between relative thalamic volume and both the difference between infants with c-PVL and controls and the interaction with PMA (left *P* = .02 and *P* = 4.7*10^−4^, right *P* = .05 and *P* = .001). When relative thalamic volumes were separated according to time of scan, the early imaging data showed significant differences (left *P* = .01 and *P* = .009, right *P* = .03 and *P* = .02). Highly significant differences were found at TEA between cases and controls (left *P* = 1.5*10^−15^, right *P* ≤ 2.0*10^−16^), but the interaction between PMA and the factor case/control was no longer significant. For infants with serial data (n = 23, 12 cases, 11 controls), a significant difference in increase of the thalamic volume was demonstrated for both the left and right thalamus between patients with c-PVL and controls (left *P* = .002, right *P* = 5.14*10^−4^). For 3 infants with c-PVL, a decrease in absolute volumes was noted with increasing PMA. The correlation between thalamic volumes and the severity of CP is shown. Infants with severe CP (GMFCS level III-V) had significantly smaller thalamic volumes compared with those with no or mild CP (absolute volumes left *P* = .02, right *P* = .03; relative volumes left *P* = .02, right *P* = .04) (Figure 4, C and D). Infants with c-PVL that developed epilepsy had significantly smaller relative thalamic volumes (left *P* = .02, right *P* = .04) compared with those who did not.

In infancy, data of 4 controls (one of whom was scanned twice) and 2 infants with c-PVL were available for analysis. Absolute and relative thalamic volumes were mean 6517 mm^3^ for cases vs 10 518 mm^3^ for controls, and relative thalamic volumes were 0.0035 for cases vs 0.0058 for controls. These data are in line with the results from the neonatal period.

## Discussion

We have identified differences in both FA values of the CST and thalamic volumes in the neonatal period between preterm infants with c-PVL and preterm control infants. In the CST, a significant difference in FA values was observed prior to TEA, which persisted at TEA and in infancy. The differences in absolute thalamic volumes between c-PVL and control groups were not significant in the early neonatal period but became highly significant at TEA, suggesting an ongoing process of disturbed brain development.

Previous studies have shown the CST to be affected in children with CP during childhood and adolescence.^8-10,26^ This held true for both children with c-PVL and for children with CP because of other causes, such as hypoxic-ischemic encephalopathy, suggesting that the damage to the CST can be caused by different mechanisms. The current study confirms the effect of extensive white matter damage on the CST at a very early stage. This effect can be found soon after the insult, as the difference in FA values, both in the region of the PLIC and in the entire CST, was already present on imaging obtained within the first weeks after the insult in most patients. This suggests acute damage to the white matter as a result of the ischemic and inflammatory processes occurring in c-PVL.^27^ This damage to the white matter persisted, as shown in the lower FA values at TEA and in infancy. The lasting effect of damage to the CST is likely to result in impaired gross motor outcome in most infants. Indeed, our data in infancy suggest that maturation of the CST does not completely stop in infants with c-PVL, but continues at a reduced rate and will not reach the values found in healthy infants of the same age. Murakami et al have previously shown that FA values in the CST below 0.5 predict the development of CP in children ranging from 9-41 months of age.^28^ All infants with c-PVL who were imaged in infancy or early childhood in this study developed CP and, except for 1 infant with a mild CP (GMFCS level I), FA values of the CST were indeed below 0.5.

In contrast to the CST, absolute thalamic volumes were not significantly different between cases and controls on the early MRI prior to TEA. They were, however, highly significant on the TEA scan, both in absolute volumes as well as after correction for overall brain size. This suggests a more remote effect of injury to the thalamus, which develops over time. The limited but significant difference between cases and controls in relative thalamic volume at the early scan suggests that this process starts directly after the injury. White matter damage in c-PVL leads to axonal disturbances, which in turn will have an effect on thalamic development. Impaired maturation of the late oligodendrocyte progenitors, known to be especially susceptible to ischemic damage, leads to a failure in myelination.^29^ Afferent and efferent axons between the thalamus and cortex as well as the thalamus and the brain stem and cerebellum may be affected, thereby, impairing normal development of the connections that are being formed during the last trimester of pregnancy.^27^ Alternatively, neuronal loss and gliosis may directly cause thalamic atrophy because of impaired input to the thalamus.^27^ Our findings are in agreement with histologic studies that have shown extensive damage to the thalamus in infants with c-PVL,^30,31^ and a functional MRI study showing diminished thalamic connectivity to the caudate nucleus, cingulate cortex, and cerebellum.^8^ Smaller thalamic volumes in infants with c-PVL were found previously on ultrasound 21 days after birth and on MRI performed in infancy, and these seem to relate to both motor and cognitive outcome.^12-14,32,33^ The decrease in thalamus/TBV between the early and TEA scan in the infants with c-PVL in this study, and for some infants even a decrease in absolute thalamus volumes, suggest that the remote effects develop over time as a result of impaired input to the thalamus because of extensive white matter damage.^27^ Thalamic volumes were related to gross motor outcome and the development of epilepsy, as those infants that developed severe CP (GMFCS level III-V) and/or epilepsy had smaller thalamic volumes at TEA.

Brain injury caused by c-PVL is extensive and involves more structures than just the CST and the thalamus. Many other brain structures have previously been shown to be affected, such as the corpus callosum, optic radiation, thalamic radiation, and sensory tracts.^26,34-36^ The combination of deficits seen in survivors of c-PVL is, therefore, likely caused by a global pattern of injury. The fact that thalamic volumes, but not FA values of the CST predicted the severity of CP in the current study suggests that the extent of the lesions, likely reflected in the thalamic volumes, is an important contributor to gross motor outcome. However, not all infants with c-PVL will develop motor impairment. The appearance of myelination of the PLIC at TEA was highly predictive of subsequent motor performance, similar to previous reports.^6,37^

Most infants with c-PVL were born after a gestation of more than 28 weeks, with a median GA of 30 weeks. Rather than extreme prematurity, factors such as infection and inflammation seem the most likely cause of the extensive brain damage in these infants.^38,39^ The majority of controls in the present study, however, were extremely preterm infants, with a lower GA than the infants with c-PVL, as only infants with a GA below 28 weeks had routine MRI at TEA in Utrecht. Both FA values and volumetric measurements can be affected by preterm birth. When compared with healthy, term born infants, preterm infants at TEA generally show lower FA values and smaller volumes, although this is influenced by the severity of illness during the neonatal period.^40-43^ The difference between infants with c-PVL and healthy, term born controls is, therefore, likely to be even larger than shown in this study. Because of ethical regulations, we were unable to scan a sufficient number of healthy control infants, specifically from Utrecht. We do, however, believe that by selecting only those infants without overt brain injury and with a normal motor outcome, controls represent infants with a likely normal or approaching normal, brain development.

There were several limitations to this study. First, as this was a retrospective study using data acquired over a number of years, different DTI protocols were used. FA values were, therefore, not comparable between all cases and controls, although the pattern of FA decrease remained. As data were acquired at 2 sites, different MR scanners and head coils were also used. Second, as described above, all control infants were born prematurely, and this may have affected their brain development as well. However, if anything, their FA measurements would have been lower compared with term born infants and the results of this study may, thus, underestimate but not overestimate the differences between infants with c-PVL and controls. Third, some of the infants with c-PVL were still very young, and the severity of their CP could not yet be determined or may change as they grow older. However, a previous study showed that the majority of infants with c-PVL grade III will develop severe CP (GMFCS level III-IV), and this will remain stable during childhood.^44^

In conclusion, the injury to the CST in infants with c-PVL is already detectable on early MRI scans, well before TEA, whereas the injury to the thalamus develops in the weeks between birth and TEA. This may reflect the difference between acute and remote effects of extensive injury to the white matter. These findings suggest that measuring the remote effects at TEA is necessary to assess any effects from future medical interventions initiated after the diagnosis of c-PVL. More long-term outcome data after advanced neonatal imaging may further elucidate the underlying mechanisms causing neurodevelopmental impairment in these infants.

## Figures and Tables

**Table II:** Available MRI data

	Utrecht		London	
	Cases (n = 26)	Controls (n = 21)	Cases (n = 7)	Controls (n = 10)
DTI				
Early	12	10	3	4
TEA	19	18	6	10
Infancy	7	0	4	3
Early + TEA	7	7	1	2
Early + infancy	1	0	0	0
TEA + infancy	5	0	2	1
Early + TEA + infancy	1	0	2	2
Volumes				
Early	14	10	3	4
TEA	21	18	7	10
Early + TEA	9	7	3	4

For the cases from Utrecht, DTI sequences were as follows: early scan 6 infants with the 3T 32 directions protocol, 4 infants with the 3T 45 directions protocol, and 2 infants scanned at 1.5T; and TEA scan 10 infants with the 3T 32 directions protocol, 7 infants with the 3T 45 directions protocol, and 2 infants scanned at 1.5T. All control infants were scanned with the 3T 32 directions protocol.

**Table I:** Patient characteristics

Characteristics	Infants with c-PVL (n = 33)	Control infants (n = 31)	*P* value
GA (wk, median [range])	30.1 [24.7-35.4]	27.9 [25.6-31.0]	<.001
Sex (M/F)	24/9	19/12	.27
PMA early scan (wk, mean [range])	33.7 [29.3-38.0]	31.3 [30.3-34.7]	.01
PMA TEA scan (wk, median [range])	41.1 [38.0-43.7]	41.4 [39.3-43.3]	.50
PMA late scan (mo, median [range])	31.4 [21.2-76.2]	26.2 [26.2-34.2]	.38
Birth weight (g, mean [range])	1621 [705-2780]	1083 [565-1630]	<.001
Prolonged premature rupture of membranes (number [%])	5 [8]	4 [9]	.80
Blood culture proven sepsis (number [%])	6 [10]	7 [16]	.67
Mechanical ventilation >7 d (number [%])	5 [8]	3 [7]	.51

*F*, female; *M*, male.
